# Photophysiological and transcriptomic response to the broad-spectrum herbicides atrazine and glyphosate in a photosynthetic picoeukaryote

**DOI:** 10.1099/mgen.0.001402

**Published:** 2025-06-25

**Authors:** Patrick A. da Roza, Thomas R. Collier, Hugh D. Goold, Sasha G. Tetu, Ian T. Paulsen

**Affiliations:** 1 ARC Centre of Excellence in Synthetic Biology, Macquarie University, Sydney, NSW 2109, Australia; 2 School of Natural Sciences, Macquarie University, Sydney, Australia; 3 New South Wales Department of Primary Industries, Orange, NSW 2800, Australia

**Keywords:** atrazine, glyphosate, photophysiology, *Picochlorum *sp., SENEW3, transcriptomics

## Abstract

To feed the growing global population, intensive agriculture relies on herbicides to maximize productivity, but these come with broad-reaching environmental impacts, particularly deleterious effects on ecosystems through water run-off systems. Picoeukaryotes, with minimal genomes, can be employed to model the modes of action of herbicides on off-target species in the environment. *Picochlorum* sp. SENEW3 (*P*. SENEW3) is a poikilohaline green alga and serves as a useful model picoeukaryote due to its small genome and robust growth characteristics. Here, we examined the growth, photophysiological and transcriptomic responses of *P*. SENEW3 to sublethal concentrations of two common herbicides: atrazine and glyphosate. Atrazine treatment resulted in significant (*P*<0.0001) reductions in mean photosynthetic maximum yield (*F_v_*/*F_m_*) of 46% and an increase in fluorescence minimum (*F_o_*) of 83%, while glyphosate treatment resulted in a significant (*P*<0.0001) 30% mean reduction in fluorescence maximum (*F_m_*). Atrazine treatment resulted in significant transcriptomic changes (*P*<0.01, 1.5 log2 fold change ratio), with increased transcription of 18 genes largely involved in gene expression and ribosomal subunits, carotenoid biosynthesis, carbon fixation and reduced transcription of 27 genes largely related to DNA replication and cell cycle. Treatment with glyphosate resulted in increased transcript abundance of 45 genes, most notably those related to energy generation and redox, and chloroplastic non-photochemical quenching and reduced transcription of 188 genes, largely involved in aa and protein synthesis. Both herbicides resulted in a reduced abundance of transcripts for a nitrogen assimilation cluster. These results highlight the potential for commonly used herbicides to have adverse effects on coastal primary producers and demonstrate the value of *P*. SENEW3 as a robust model for evaluating the impacts of horticultural compounds and agricultural practices on this ecologically important group of organisms.

## Graphical abstract

Summary of *P*. SENEW3 photophysiology and transcriptomic changes in response to atrazine and glyphosate. Created using biorender.com.

**Impact Statement**

The poikilohaline photosynthetic eukaryote *Picochlorum* is an emerging model for the study of algal cell biology and genetics. These microbes can be used as a model for plant genetics and cell biology and also offer an opportunity to study the broad physical environments they exist in. As global agriculture intensifies, so does the application and use of fertilizers and herbicides. Little is known about how herbicides affect aquatic organisms and their marine and estuarine trophic chains, and the modes of action of herbicides on plant cells are incomplete. This article presents a first clear look at the effects of sublethal concentrations of two widely used herbicides, glyphosate and atrazine, on the transcriptome of *Picochlorum*. This work demonstrates differing responses to different herbicides, for example, an oxidative stress response to atrazine exposure and a photoprotection response on exposure to glyphosate, and highlights a common physiological response, repression of nitrogen assimilation. This data highlights the value of a simple and relatively non-redundant plant-like genome for precise genetic dissection of responses of the plant cell to stressors.

## Data Summary

RNA-Seq data is available through the NCBI SRA, with accession numbers SRR24234282 to SRR24234290 under BioProject PRJNA839791 and BioSample SAMN28555558. Gene transcription levels, differential abundance and pulsed amplitude modulation measurements are available with the provided supplementary data.

## Introduction

The global population in 2021 is nearing eight billion and is continuing to grow; however, current agricultural practices have remained largely unchanged since the third agricultural revolution of the 1950s and 1960s [1]. The impacts of this population growth, coupled with climate change and loss of wild space, now require a drastic change in agricultural systems. A key anthropogenic pollutant, pesticides, has seen continual increasing use. In 2021, global agricultural pesticide application reached 3.5 million tonnes [2] and a total global trade of 7.0 million tonnes [3], the majority of which is herbicide [4,6]. This overzealous use of herbicides has resulted in numerous instances of environmental degradation [7] and the development of resistant weed biotypes [4,8,11].

Atrazine [6-chloro-N^2^-ethyl-N^4^-(propan-2-yl)−1,3,5-triazine-2,4-diamine] and glyphosate [*N*-(phosphonomethyl) glycine (as an isopropylamine salt)] are the two most widely used herbicides in the world [12,14]. Atrazine modifies photosynthesis, growth and enzymatic activity in photosynthetic organisms by competitively binding the plastoquinone-binding protein of photosystem II (PSII), thereby blocking electron transport within the chloroplast photosystems. This inhibits photosynthesis and is thought to induce plant death through photo-oxidative damage of chlorophyll proteins caused by the formation of oxygen radicals [15]. Glyphosate’s primary herbicidal mechanism is the disruption of the shikimate pathway by inhibition of 5-enolpyruvylshikimate-3-phosphate synthase (EPSP), preventing the biosynthesis of aromatic aa (phenylalanine, tyrosine and tryptophan) in plants and green algae [16,17]. In this way, glyphosate affects photosynthesis indirectly, inhibiting the biosynthesis of chlorophyll, carotenoids, fatty acids and other secondary metabolites [18].

Previous toxicological studies have investigated the effects of either atrazine or glyphosate on green algae [19,21]. However, few studies have examined the transcriptional response of green alga to these toxicants. Transcriptional responses of the model unicellular green alga *Chlamydomonas reinhardtii* focused on the impacts of sublethal atrazine exposure [22,23], demonstrating that short-term exposure to atrazine can result in increased transcription of genes related to electron disposal and heterotrophic energy production [22]. Atrazine was also shown to decrease transcripts associated with photosynthesis, including those related to light harvesting, chlorophyll binding and carbon concentration mechanisms [23]. However, *C. reinhardtii* is a known mixotroph. While this has been particularly useful for the study of photosynthesis and carbon metabolism [24,25], it may not necessarily reflect the response of obligate phototrophs, indicating a need to consider impacts on a wider range of green algae.

*Picochlorum* sp. SENEW3 (*P*. SENEW3) is a poikilohaline photosynthetic picoeukaryote green alga (phylum: Chlorophyta) with burgeoning potential in genomic and biotechnological applications. *P*. SENEW3 displays robust growth characteristics under fluctuating environmental conditions [26]. Little work, however, has been undertaken in this organism in regard to gene expression, with transcriptomic work so far only considering variations in salinity and temperature [27,31]. As an exemplar of a coastal marine (lagoon) green algae, *P*. SENEW3 represents a group of organisms likely to be impacted by human activities, especially by herbicides leaching from agricultural run-off.

To investigate the response of *P.* SENEW3 to sublethal treatments of atrazine and glyphosate, growth inhibition was assessed through flow-cytometric population cell counts, photophysiology via pulsed amplitude modulation (PAM) fluorescence and transcriptomic responses via RNA-Seq. This work demonstrates the use of this relatively fast-growing photosynthetic eukaryote in the rapid assessment of cellular and transcriptomic impacts of agricultural compounds on photosynthetic organisms. Such approaches will facilitate the investigation of the impacts of other herbicides and pesticides in agriculture and aquaculture, informing future use in the broader context of increasing crop productivity while concurrently balancing human and environmental health.

## Methods

### *P.* SENEW3 cell culture

*P*. SENEW3 was obtained from Professor Brian Palenik at Scripps Institution of Oceanography, University of California at San Diego, CA, USA [26], and was cultured in f/2 medium (without sodium silicate) as described by Guillard and Ryther [32,33] with Keller (K) medium artificial seawater base [34]. All cultures were grown at 22 °C, 120 r.p.m. (Infors HT Multitron incubator), with light emitting diodes (LED) cool white irradiance at 100 µmol photons m^−2^ s^−1^ on a 14 h light: 10 h dark cycle; maintenance cultures were transferred weekly to fresh medium. Cultures were periodically treated with an antibiotic mixture (50 µg ml^−1^ ampicillin, 10 µg ml^−1^ gentamicin, 20 µg ml^−1^ kanamycin and 100 µg ml^−1^ neomycin) [35] and/or aseptically cell sorted with a BD Influx flow cytometer to prevent bacterial or fungal contamination. Cell counts for culture passaging and inoculation were performed with a ‘Neubauer improved bright-line’ haemocytometer slide with an Olympus BH-2 microscope as per the manufacturer’s instructions.

### Flow cytometry

Atrazine [6-chloro-N^2^-ethyl-N^4^-(propan-2-yl)−1,3,5-triazine-2,4-diamine, Sigma-Aldrich 49085–100 mg] stocks (5 mM in methanol) and glyphosate [*N*-(phosphonomethyl) glycine, Sigma-Aldrich P9556-5G] stocks (60 mM in H_2_O) were filter sterilized through a 0.2 µm pore size membrane, aliquoted and stored at −20 °C. *P*. SENEW3 were inoculated in 8 ml f/2 media at ~4×10^5^ cells ml^−1^ in Greiner clear 6× multiwall plates. Cultures were treated with the addition of atrazine (0.00 µM, 0.05–5.0 µM final conc.) or glyphosate (0 mM, 0.6–6.0 mM final conc.) in biological triplicates and incubated for 2 weeks. Flow cytometry (FCM) cell counts were assessed via a BD Accuri C6 flow cytometer as events µl^−1^. Polyscience microspheres (1.0 µm) (Polysciences, Inc. NC9862439) were used as an event rate control. Cells were gated via forward scatter area (FSC-A)/side scatter-area and FSC-A/PercP-A (Ex 488 nm, Em 670 nm long pass). Population density and high chlorophyll fluorescence intensity were utilized as indicators of cell viability (Fig. S1, available in the online Supplementary Material).

### PAM fluorometry

*P*. SENEW3 photosynthetic maximum quantum efficiency response to herbicide treatment was assessed using a Heinz Walz PHYTO-PAM Phytoplankton Analyzer. Briefly, 150 ml cultures of *P*. SENEW3 were inoculated at a density of ~1×10^7^ cells ml^−1^ (haemocytometer) and supplemented with 0.25 µM atrazine, 2.5 mM glyphosate or no herbicide (control) in biological triplicate. Aliquots were taken from each sample at time points 3, 24, 48 and 72 h (light/day cycle), incubated in the dark for a minimum of 15 min and transferred to a quartz cuvette and placed within a PHYTO-ED Emitter-Detector unit. The emission of mean fluorescence minimum (*F_o_*) was determined under modulated light (470 nm for green algal cells), and mean fluorescence maximum (*F_m_*) was determined by the application of a saturating pulse of the same wavelength in triplicate, 30 s apart. Maximum quantum efficiency/yield of PSII photochemistry (*F_v_*/*F_m_*) was calculated as *F_v_*/*F_m_*=[*F_m_*−*F_o_*]/*F_m_* [36].

### Statistical analysis

FCM growth data was analysed using one-way ANOVA followed by Dunnett’s multiple comparison test of day 7 cell counts with a 95% confidence interval. All herbicide treatment concentration data was compared with no-treatment controls at matching timepoints and considered significant (alpha) at *P*<0.05. Photophysiology data (mean *F_v_*/*F_m_*, *F_o_* and *F_m_*) was analysed using two-way repeated measures ANOVA followed by Tukey’s multiple comparison test for the main treatment (atrazine, glyphosate and control) effect, with pooled variance across time (3, 24, 48 and 72 h) and significance set at (alpha) *P*<0.05. All statistical analyses were conducted using GraphPad Prism v8 for Windows (GraphPad Software, San Diego, CA, USA).

### Transcriptomics

RNA-Seq was utilized to assess the transcriptomic response of *P*. SENEW3 to atrazine and glyphosate. *P*. SENEW3 cultures were grown to early to mid-exponential growth phase (~1×10^7^ cells µl^−1^) and brought to a final concentration of 0.25 µM atrazine or 2.5 mM glyphosate along with no-treatment controls in biological triplicate. Cultures were incubated at 22 °C, 120 r.p.m. at 100 µmol photons m^−2^ s^−1^ for 3 h. Samples were centrifuged at 3,200 ***g*** for 15 min at 4 °C with 0.1% Pluronic F-68 (Sigma-Aldrich P1300). Pellets were resuspended in 700 µl of TRIzol™ reagent (Thermo Fisher Scientific, catalogue number: 15596026), homogenized and transferred to 2 ml BeadBug™ prefilled tubes with 0.1 mm acid washed silica glass beads (Sigma-Aldrich: Z7637621-50EA). Samples were snap frozen with liquid nitrogen and stored at −80 °C.

*P*. SENEW3 frozen pellets were lysed and homogenized via bead beating using a Precellys Evolution tissue homogenizer (Bertin Instruments) at 7,200 r.p.m. (3×30-s homogenization, 30-s rest intervals, at 4 °C). Total RNA was extracted using the Qiagen miRNeasy Mini Kit (Cat. No./ID: 217004) with residual DNA digestion using Qiagen RNase-Free DNase Set (Cat No./ID: 79254) and eluted with RNase-free water. Sample quality and concentration were determined via NanoDrop RNA analysis and Qubit™ RNA HS Assay Kit (Invitrogen Q32855). Samples underwent RNA-Seq analysis (Illumina stranded mRNA, NextSeq 2×75 bp MID sequencing) at the Ramaciotti Centre for Genomics, NSW, Australia.

FastQ RNA-Seq reads were imported into Geneious Prime software (v2021), adapter trimmed, paired and mapped to the *P*. SENEW3 genome (NCBI BioProject: PRJNA839791, accession: CP120453 - CP120466) [37] using Geneious RNA-Seq mapper (default setting). Mapped reads were used to calculate expression levels and were normalized as the number of transcripts per million for each replicate [38]. DESeq2 (parametric) [39] was used for the comparison of transcript expression between atrazine or glyphosate and controls. Adjusted *P*-value was set to *P*<0.01, and log2 fold change ratio (log2 FCR) was examined for up- and downregulated gene transcription. Functional categories of significantly altered transcripts were manually assigned based on genomic annotation (blastp [40] and InterProScan [41]), KEGG GhostKOALA [42] and eggnog-mapper v2 [43,44] assignments using the categorization scheme of Hemschemeier *et al*. [45].

## Results and discussion

*P*. SENEW3 exhibits different physiological responses against atrazine and glyphosate, two of the world’s most intensively applied herbicides. *P*. SENEW3 was initially examined for growth inhibition to both atrazine and glyphosate and subsequently exposed to sublethal concentrations of 0.25 µM and 2.5 mM, respectively, for assessment of photophysiological and transcriptomic responses. Both atrazine and glyphosate have previously been shown to have a wide range of effects in photosynthetic organisms. In general, atrazine treatment is known to result in high levels of oxidative stress due to the generation of reactive oxygen species (ROS) including singlet oxygen, hydrogen peroxide, hydroxyl radicals and superoxide anion radicals [46]. Glyphosate, however, typically interferes with aromatic aa synthesis due to its inhibition of the shikimate pathway and subsequently disruption of chlorophyll production, photosynthesis, respiration and generation of ROS [12,47, 48].

### Growth inhibition

**Fig. 1.**
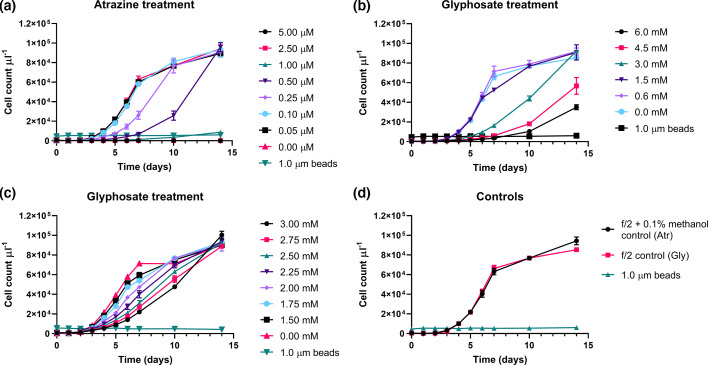
Herbicide growth inhibition curves for *P*. SENEW3 over 14 days in f/2 medium at 22 °C, 120 r.p.m., 100 µmol m^−2^ s^−1^ white light (14 h light: 10 h dark). Mean cell counts of biological triplicates estimated by the number of population events µl^−1^, sampling a total of 14 µl per sample using BD Accuri C6 flow cytometer. (a) Population cell counts µl^−1^ for 0 to 5 µM atrazine treatment. (b) Population cell counts µl^−1^ for 0 to 6 mM glyphosate treatments. (c) Population cell counts µl^−1^ for 0 to 3 mM glyphosate treatments. (d) Population cell/event counts µl^−1^ for control treatment groups.

*P*. SENEW3 growth inhibition in the presence of a range of atrazine and glyphosate concentrations was used to assess appropriate sublethal treatment concentrations for photophysiology and transcriptomic experiments (Fig. 1). While susceptibility can vary greatly, phytoplankton (including photosynthetic cyanobacteria and algae eukaryotic protists) are some of the most sensitive aquatic organisms to herbicidal exposure, particularly the triazine class of herbicides such as atrazine [20,46, 49, 50].

Comparison of treatment and control flow cytometric counts at day 7 showed significant differences (*P*<0.0001) for atrazine concentrations of 0.25 µM and above and glyphosate concentrations of 2 mM and higher (Tables S1 and S2). Exposure to 0.25 µM atrazine resulted in a 58% population reduction compared with no-treatment controls, while 2.5 mM glyphosate resulted in a 57% population reduction. This effective concentration of atrazine was similar to the EC50 (0.25 µM) employed by Esperanza *et al*. [22] in examining the effect of atrazine on the model green alga *C. reinhardtii*. Notably, atrazine appeared to be highly cytotoxic in comparison with glyphosate, with atrazine resulting in similar growth inhibition at 10,000-fold lower molar concentration.

These results are not unexpected as glyphosate itself is known to be one of the least toxic herbicides [51], and past work has shown it to be significantly less cytotoxic than atrazine in the green algae *Raphidocelis subcapitata* [49]. Commercial glyphosate formulations, however, are known to possess greater toxicity due to the inclusion of surfactants such as polyethoxylated tallow amine, which is markedly more lethal than glyphosate itself [52]. Given this, a focus for future investigations would be investigating the toxicity of common formulation surfactants (detergents, emulsifiers, dispersants and wetting and foaming agents) on *P*. SENEW3*,* to look at their potential contributions to herbicide exposure impacts in the field.

### Photophysiology

The use of PAM chlorophyll fluorescence to monitor the photosynthetic activity of both plants and algae is well characterized, enabling assessment of PSII photochemistry, photosystem electron flux and carbon assimilation [36,50, 53]. Thus, PAM fluorometry was used to determine whether atrazine and glyphosate would alter the photochemical efficiency of PSII (Φ_PSII_) and thus affect photosynthesis in *P*. SENEW3 (Table S10). Tukey’s multiple comparison test was used to compare the main treatment (herbicide) effect between each of the three conditions (with pooled variance across time points (Table S3).

**Fig. 2.**
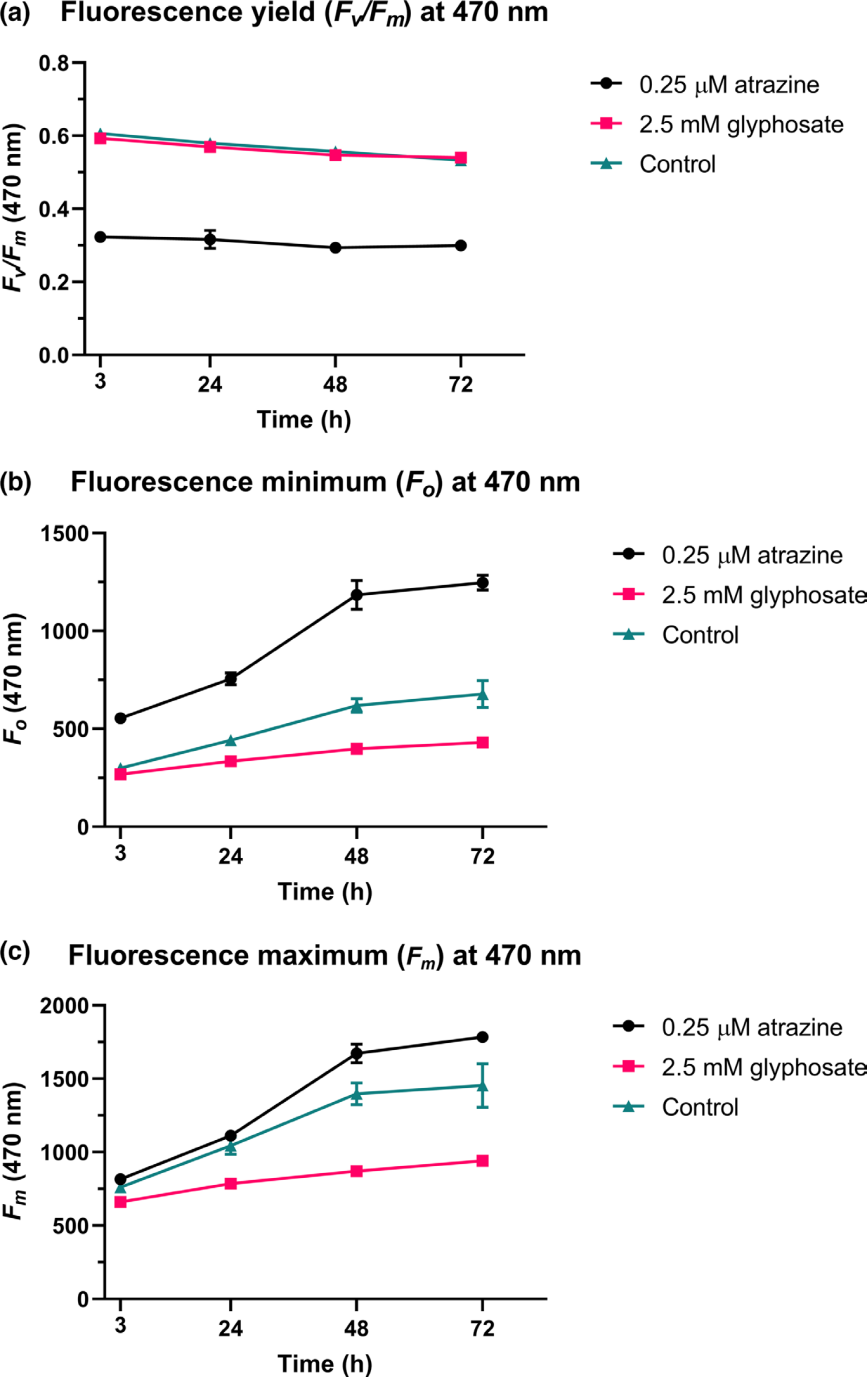
Sublethal herbicide-induced photophysiological response in *P*. SENEW3. Mean PAM fluorescence measurements at ex 470 nm for 0.25 µM atrazine, 2.5 mM glyphosate and control (no herbicide) treatment groups at 3, 24, 48 and 72 h. Each biological triplicate was measured in technical triplicate 30 s apart, after at least 15 min of dark adaptation. Data points display sample mean and sd. (a) Maximum fluorescence yield (*F*_*v*_/*F*_*m*_). (b) Fluorescence minimum (*F*_*o*_). (c) Fluorescence maximum (*F*_*m*_).

Treatment with 0.25 µM atrazine resulted in a significant (*P*<0.0001) 46% mean reduction in the maximum quantum yield of PSII (*F_v_*/*F_m_*) and 83% mean increase in fluorescence minimum (*F_o_*), as well as a slight increase in fluorescence maximum (*F_m_*) of 16% (*P*=0.0049) compared with controls (Fig. 2 and Table S3). Measurement of PSII *F_v_*/*F_m_* is frequently used as an indicator of abiotic stress, and past work has demonstrated reduced PSII quantum yield in the green alga *R. subcapitata* when exposed to atrazine [54]. Increases in the *F_o_* are instead associated with oxidative damage and loss of PSII reaction centres [55,56], which can result from photoinactivation [57]. For example, heat stress has been shown to increase *F_o_* in barley leaves [58].

In contrast, 2.5 mM glyphosate treatment resulted in no significant change in *F_v_*/*F_m_* but did result in a mean decrease of 30% in both *F_o_* (*P*=0.0011) and *F_m_* (*P*=0.0001) compared with controls. Reductions in *F_m_* are known to decrease as a result of photosynthetic tissue stress and subsequent increase of non-photochemical quenching (NPQ); which acts by dissipating excess excitation energy as thermal radiation (heat) as thermal radiation (heat) [59]. These changes in *F_v_*/*F_m_*, *F_o_* and *F_m_* indicate the occurrence of strong photophysiological stress in *P*. SENEW3 in the presence of atrazine and a possible NPQ stress response in the presence of glyphosate.

### Transcriptomic data analysis and broad-scale changes

**Fig. 3.**
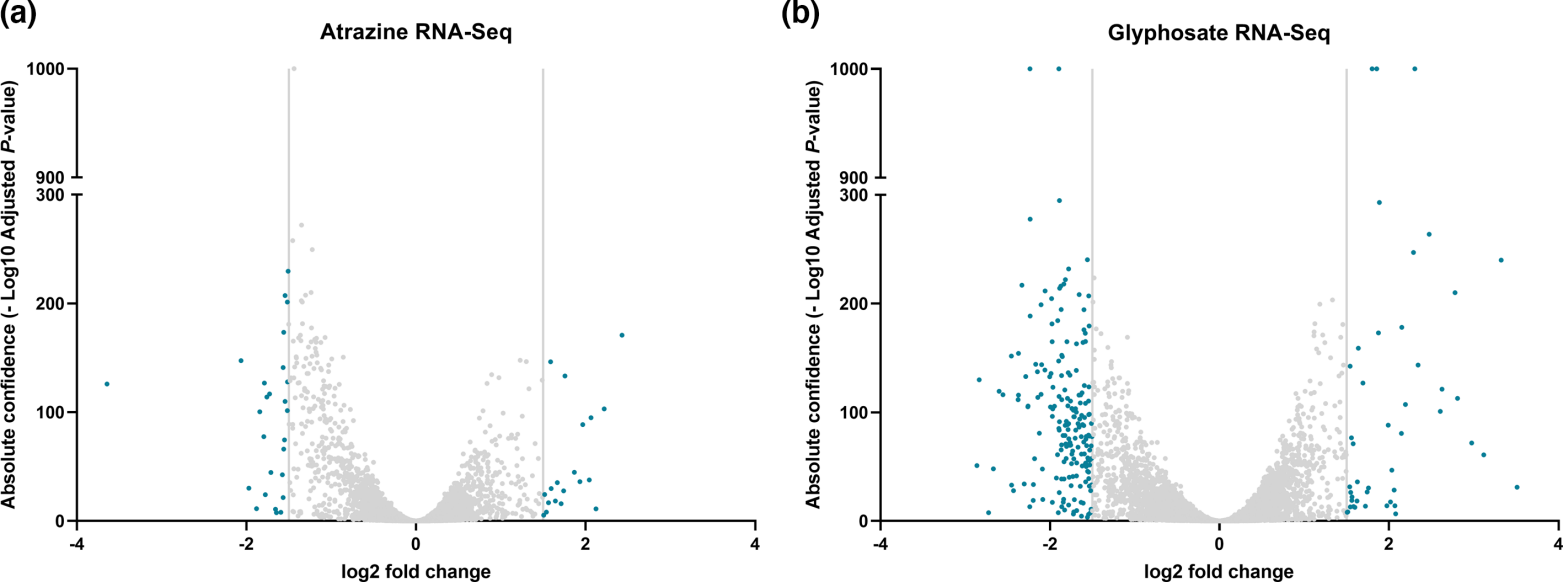
Volcano plots of transcriptomic DESeq2 analysis. Statistically significant data points/genes (adjusted *P*<0.01) displayed in teal/blue, log2 FCR cut-off set to ±1.5. (a) 0.25 µM atrazine treatment group vs. control and (b) 2.5 mM glyphosate treatment group vs. control.

Transcriptomic analysis of atrazine- and glyphosate-treated cell cultures was undertaken via RNA-Seq. Reads were mapped to the *P*. SENEW3 genome [37] (Tables S8 and S9) and assessed for differential gene expression between each treatment group (atrazine or glyphosate) and the control (no treatment) with DESeq2. Atrazine treatment resulted in an increased transcriptional abundance of 18 genes and a reduced abundance of 27 genes, while glyphosate treatment resulted in 45 genes increased and 188 genes decreased in transcriptional abundance, respectively (*P*<0.01 significance and ±1.5 log2 fold change cutoff, Fig. 3).

**Fig. 4.**
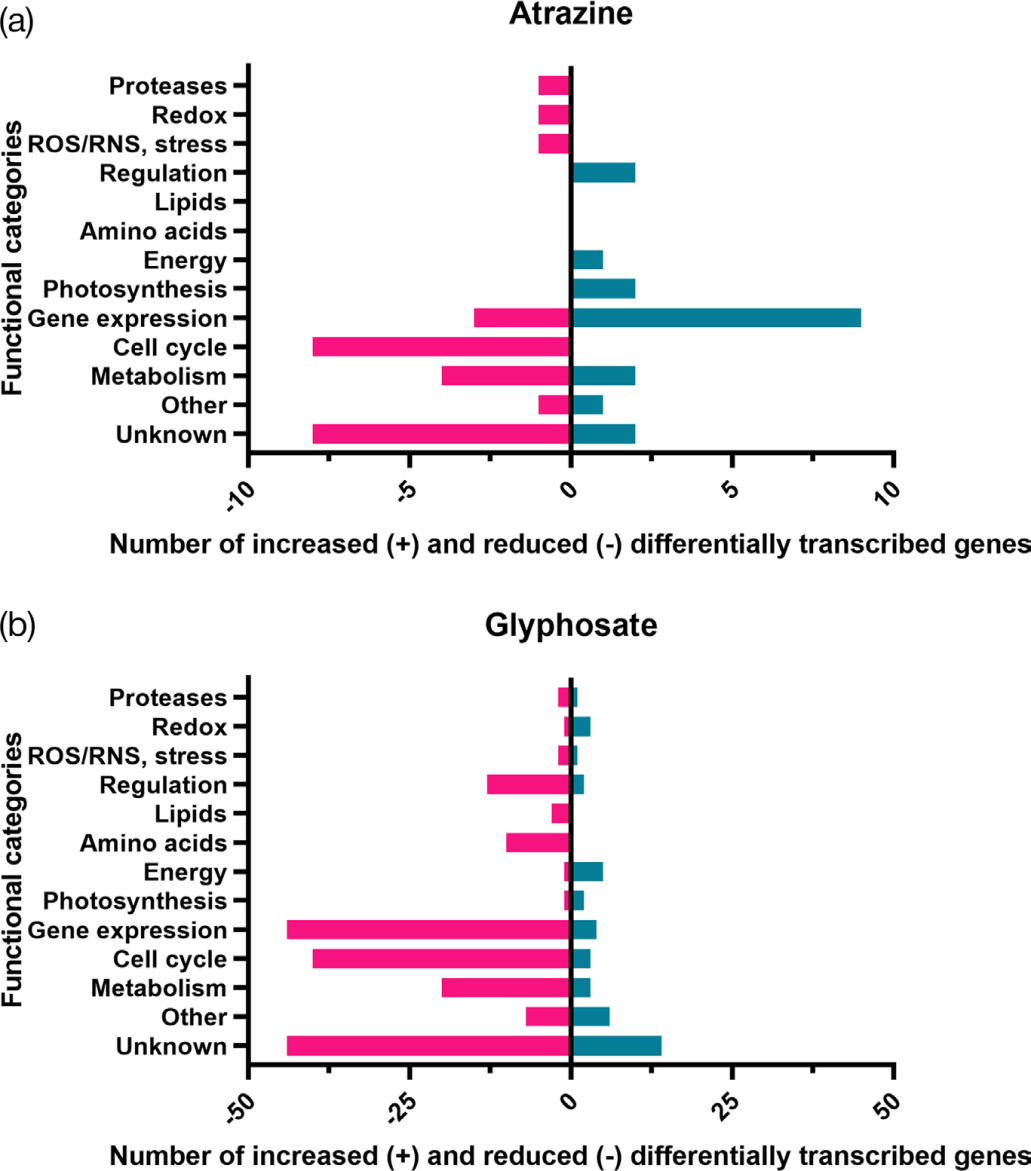
Differentially transcribed genes assigned to functional categories. Number of significant (adjusted *P*<0.01) differentially expressed genes with increased (log2 FCR ≥1.5) or decreased (log2 FCR ≤1.5) transcript abundance, assigned to functional categories for (a) 0.25 µM atrazine and (b) 2.5 mM glyphosate treatments, against negative controls.

These sets of strongly differentially transcribed genes were manually assigned to functional categories based off genome, KEGG and EggNogg annotations using the scheme previously developed by Hemschemeier *et al*. [45] (Table S7), to determine what functions were most strongly impacted by each treatment (Fig. 4). Atrazine treatment resulted in increased transcription of genes largely related to gene expression (9 of 18), as well as reduced transcription of genes associated with cell cycle processes (8 of 27). In general, the transcriptional response to glyphosate treatment involved a larger and broader range of genes than atrazine. Genes highly transcribed in response to glyphosate were predominantly involved in energy, metabolism, gene expression, cell cycle and photosynthesis, whereas transcripts with reduced abundance were related to gene expression (particularly ribosomal subunits), cell cycle, metabolism (transporters and nt biosynthesis), regulation and aa biosynthesis (Table S5).

**Fig. 5.**
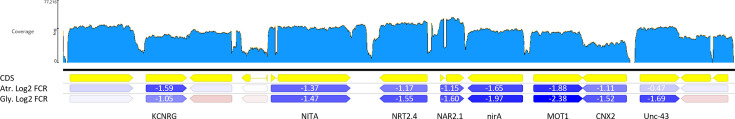
Transcription of a nitrate assimilation gene cluster. Displaying gene co-localization, coding sequence (CDS) in yellow, as well as atrazine (Atr.) and glyphosate (Gly.) gene transcription log2 FCR. Log gene transcript coverage (Geneious RNA-Seq mapper) of triplicate control samples displayed in blue above gene annotations. Gene map generated using Geneious Prime v2021 software.

Shared transcriptional changes were also examined to assess whether the two herbicides elicited any common stress responses despite differing mechanisms of action (Fig. 5). Atrazine and glyphosate treatments shared the transcriptional enhancement of 5 genes and lowered transcriptional expression of 19 genes at a ±1.5 log2 FCR cut-off (Table S4). The analysis was then extended by relaxing the fold change cut-off to include significant (*P*<0.01) transcriptional changes and all log2 FCR values. This revealed the presence of a nuclear genome nitrate gene cluster, consisting of eight co-localized genes that were downregulated in both herbicide treatment groups, four of which, *NITA*, *NRT2.4*, *NAR2.1* and *nirA*, are involved in nitrate transport and assimilation, with two additional cluster genes, *MOT1* and *CNX2*, being involved in molybdate transport and molybdopterin biosynthesis, respectively (Fig. 5).

**Fig. 6.**
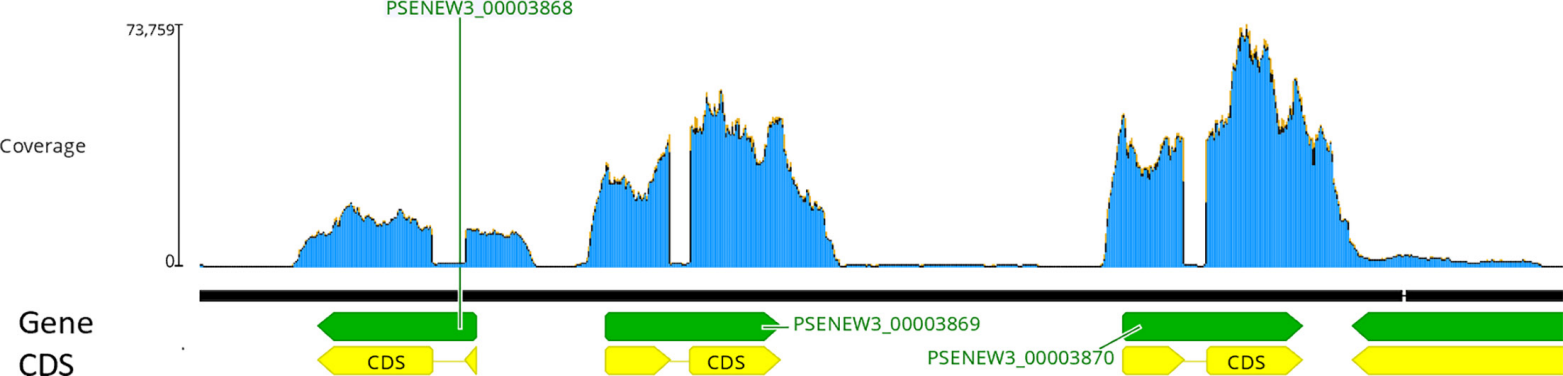
A novel highly transcribed gene cluster consisting of genes PSENEW3_00003868–3870. Displaying mapped transcript coverage (blue), gene regions (green) and protein coding sequence (CDS) regions (yellow). Gene map generated using Geneious Prime v2021 software.

In addition, gene cluster analysis led to the identification of a novel group of three co-localized genes, PSENEW3_00003868, PSENEW3_00003869 and PSENEW3_00003870 (Fig. 6), with the latter two (_3869 and _3870) sharing high aa sequence homology (73.27% shared sequence identity, Fig. S2). The three genes appear to be novel and not previously identified or characterized, as PSENEW3_00003868 only showed a very weak protein similarity (blastp) [60] to a group of fasciclin domain-containing proteins [61], while PSENEW3_00003869 and PSENEW3_00003870 had no identifiable blastp hits. Herbicide treatment did not significantly effect the transcription of these three genes, which were highly transcribed in all conditions and were, respectively, the ninth, fourth and second most abundant transcripts across the genome in the three control replicates (Table S6). Previous genomic analysis of *P.* SENEW3 has identified the existence of functional gene clusters and horizontal gene transfer candidates involved in the organisms’ robust environmental adaptions. Such unidentified gene clusters, especially those so highly transcribed, could potentially possess novel or essential functions that have been overlooked, warranting further investigation.

### Atrazine: increased protein, carotenoids and CO_2_ fixation

Transcriptomic analysis of atrazine-treated *P.* SENEW3 revealed enhanced transcription of genes involved in protein synthesis, the Calvin cycle and carotenoid biosynthesis. Increased abundance of protein synthesis (ribosomal subunits and elongation factors) gene transcripts differs from what has been reported for other algal species. For example, in *C. reinhardtii*, exposure to 0.25 µM atrazine induced an increase in the abundance of RNA-Seq transcripts mainly relating to aa catabolism [22]. The authors of the study suggested that aa degradation was utilized by *C. reinhardtii* for respiratory energy generation as they transitioned from photosynthetic to heterotrophic metabolism in order to survive. However, proteins and ribosomal RNA can be damaged by ROS, which are known to be generated under atrazine stress in photosynthetic organisms [15,62]. Furthermore, ROS species such as hydrogen peroxide (H_2_O_2_) have been observed to induce complex genome-wide regulation of protein translation, with associated increases in protein synthesis and ribosomal ORF occupancy [63,66]. Thus, the enhanced transcription of translation-related genes could represent an adaptive stress response to atrazine-induced ROS generation.

Furthermore, it was observed that atrazine exposure resulted in an increase of carotenoid biosynthesis gene transcription. This was seen in the enhanced expression of the phytoene synthase gene (*PSY1*) and, while below the set +1.5 log2 FCR cut-off, the phytoene desaturase gene (*PDS*, *P*<0.01, 1.26 log2 FCR). Together, these two enzymes catalyse the biosynthesis of zeta-carotene and form the first two committed steps of carotenoid biosynthesis [67]. It is well established that carotenoids are effective non-enzymatic antioxidants, preventing ROS damage by quenching singlet oxygen formed during photosynthesis and potentially inhibiting lipid peroxidation [68,69]. It, therefore, seems likely that carotenoid biosynthesis was enhanced in *P.* SENEW3 as a response to preventing ROS damage caused by atrazine exposure.

The other major transcriptional response in *P.* SENEW3 to atrazine was an increased abundance of transcripts involved in the Calvin cycle. This included increased transcription of genes encoding RuBisCO large subunit-binding protein subunit beta, fructose-bisphosphate aldolase (aldoc/FBPase) and sedoheptulose-1,7-bisphosphatase (CSBP/SBPase), which together catalyse the regeneration of 5-carbon sugars and ribulose-1,5-bisphosphate [70,72]. It has been demonstrated that both aldoc and CSBP modulate photosynthetic carbon flux and that their overexpression promotes CO_2_ fixation, photosynthetic rate and enhanced growth in transgenic tobacco (*Nicotiana tabacum L. cv Xanthi*) by stimulating the regeneration of ribulose-1,5-bisphosphate [70,71, 73,75]. Conversely, modulation in photorespiration, the competitive oxygenase reaction of 1,5-bisphosphate carboxylase/oxygenase [76,77], has been recognized as an important ROS stress response [78,79]. It has previously been observed in plants and algae that photorespiration is upregulated and CO_2_ fixation is downregulated under various abiotic stresses [80,83], including under atrazine exposure [84]. Photorespiratory metabolism, after recycling 2-phosphoglycerate, cycles back into the Calvin cycle [85], which may explain the increased transcription of Calvin cycle genes.

### Atrazine: reduced cell cycle and oxidases

Cell cycle and meiosis functional categories overall saw the greatest transcriptional reduction in response to atrazine treatment. Growth inhibition is one of the primary effects of herbicide application; this remains the case in their exposure to algae. Atrazine is known to cause disruption in cell division and to exert genotoxicity [46,49]. In addition, exposure of atrazine to *C. reinhardtii* in the stationary phase has been demonstrated to promote cellular senescence [86]. The lowered transcription of eight cell cycle-related genes (DNA replication and chromatin) is therefore consistent with the known effects of the herbicide.

Also observed was the reduced transcription of the gene *PSENEW3_00004722*, annotated as a respiratory burst oxidase homologue protein B (RbohD, *Solanum tuberosum*). nicotinamide adenine dinucleotide phosphate (NADPH) oxidases are involved in the generation of superoxide during ‘oxidative burst’ or ROS, mediate cellular homeostasis [87] and are often induced by pathogen infection [88]. Given that atrazine is known to disrupt cell division and generate ROS, it is reasonable to speculate that the lowered transcription of such related genes is most likely a response to limit further production of ROS and alleviate atrazine cytotoxicity.

### Glyphosate: increased glycolysis and redox

In *P.* SENEW3, we found that a major component of the transcriptomic response was the enhanced transcription of genes encoding proteins related to energy generation, most notably Aldoc/FBPase and NADP-dependent glyceraldehyde-3-phosphate dehydrogenase (GAPDH/ALDH11A3), which constitute the fourth and sixth steps of glycolysis, respectively. Alteration of glycolysis has previously been observed in glyphosate-exposed soybean, with increased transcriptional abundance of enolase (2-phosphoglycerate dehydratase), catalysing the penultimate step of glycolysis [89]. Oxidative stress has been increasingly implicated in the modification of glycolysis and tricarboxylic acid (TCA) cycle enzymes, such that redox regulation of sugar-related metabolic pathways seems to play a fundamental role in photosynthetic organisms [90]. Both GAPDH and aldoc/FBPase appear to be important in redox regulation of glycolysis and the TCA cycle, with GAPDH, being a ubiquitous house-keeping gene/protein, a target of hydrogen peroxide [91] and a known plant redox sensor [92,94].

In addition, glyphosate treatment saw increased transcription of the gene encoding mitochondrial substrate carrier family protein G′ which likely transports acylcarnitines into the mitochondrial inner membrane for fatty acid beta-oxidation [95]. A gene encoding a probable nicotinamide adenine dinucleotide (NADH)-ubiquinone oxidoreductase C3A11.07 (SPAC3A11.07), catalysing the oxidation of NADH in the mitochondrial electron transport chain during aerobic respiration [96], was also found to show increased transcription. The gene encoding for enoyl-(acyl-carrier-protein) reductase (NADH), which catalyses the final step of the fatty acid biosynthesis elongation cycle [97], was instead observed to have lowered transcription. Together, these transcriptional changes suggest an alteration of carbon flux within *P.* SENEW3 when exposed to glyphosate by modulating glycolysis, the TCA cycle and fatty acid oxidation, likely favouring the utilization of reserve energy stores.

### Glyphosate: increased zeaxanthin and NPQ

It is well documented that glyphosate exposure disrupts photosynthesis by interfering with the biosynthesis of secondary metabolites [12]. This primarily occurs via the interruption of chlorophyll biosynthesis and subsequent reduction in chlorophyll content [98,100]. Here, we saw the increased transcription of two genes/proteins involved in the photoprotection of photosynthetic complexes, namely, carotene biosynthesis-related protein (CBR) and PSII protein (PSBS). CBR is a putative zeaxanthin binding protein that is thought to play a role in photoprotection as an adaptive response to stress-induced photodamage [101], whereas PSBS2 is required for NPQ, protecting the photosynthetic apparatus from excessive photo-oxidative damage, and is known to accumulate under high light [102,103]. It has been observed that, together, the binding of zeaxanthin to PSII and the protonation of PSBS result in a conformational change in the PSII antenna complex that increases the quantum yield of NPQ [104,105]. This protects the cell from excessive photo-oxidative damage by improving the thermal dissipation of received energy. Thus, it appears that *P.* SENEW3 protects itself from glyphosate-induced photo-oxidative damage by increasing the levels of NPQ. This is consistent with our observed decrease in *F_m_* fluorescence (Fig. 2), a known consequence of increased NPQ [59].

### Glyphosate: reduced protein, aa synthesis and shikimate pathway

Gene expression was the largest functional category with decreased gene transcription in response to glyphosate treatment (Fig. 4). This is not unexpected due to the glyphosate’s disruption of aromatic aa biosynthesis (phenylalanine, tyrosine and tryptophan) by the inhibition of EPSP as the sixth reaction in the shikimate pathway [16]. Decreased transcription of protein and aa biosynthesis and shikimate pathway genes likely reflects a response to this mechanism. There was also lowered transcription of 12 gene expression-related genes, predominantly involved in the transcription and protein biosynthesis (ribosomal proteins), and an additional 8 aa acid biosynthesis genes including those related to the biosynthesis of proline (*At3g62120*); histidine (*HISN1B*); l-tryptophan (*ASA1*); leucine, isoleucine and valine (*ilvX*); l-homocysteine (*SAHH*); and l-serine and *S*-adenosyl-l-methionine (*METM*) (Table S6). Most notable was the reduced transcription of genes encoding 3-dehydroquinate dehydratase/shikimate dehydrogenase protein, which catalyses two consecutive reactions (steps three and four) of the shikimate biosynthesis pathway, converting 3-dehydroquinate first to 3-dehydroshikimate and then to shikimate [106]. These results indicate the disruption of the shikimate pathway, though not EPSP itself, as well as broader impacts on aa and protein synthesis in *P.* SENEW3 under glyphosate exposure.

### Decreased transcription of a nitrogen assimilation gene cluster

Nitrogen assimilation, photosynthesis, carbon fixation and ROS are strongly coordinated in green algae and other photosynthetic organisms [90,107, 108]. Energy and reducing power (NADPH) from photosynthesis is used for the assimilation of CO_2_ and nitrate ions (NO_3_^-^) for the formation of carbohydrates and aa, respectively. Conversely, chloroplast degradation caused by ROS can result in a pool of excess nitrogen, which is remobilized with the cell and is associated with a decrease in environmental nitrogen assimilation [109]. Here, transcriptomic analysis revealed the reduced transcription of a nitrate assimilation gene cluster in *P.* SENEW3 in response to treatment with both atrazine and glyphosate. Downregulation of nitrogen assimilation and nitrate reductase activity has previously been observed in photosynthetic organisms in response to both atrazine and glyphosate exposures. For example, nitrate reductase activity has been previously shown to be reduced in the green alga *C. reinhardtii* [22] and the cyanobacteria *Microcystis aeruginosa* [110] in response to atrazine stress, whereas nitrogen fixation/assimilation has been found to be decreased in non-resistant soybean (*Glycine max*) [111], and nitrogen reductase activity and leaf nitrogen content lowered in non-resistant corn (*Zea mays* L.) in response to glyphosate exposure [112]. Previous analysis of the *P.* SENEW3 genome has revealed the presence of multiple functional gene clusters relating to metabolic pathways. These include nitrate assimilation, urea metabolism, acetate assimilation/fermentation, glucose uptake and photorespiration, some of which have been shown to be co-expressed under salinity stress. Such functional gene clusters are likely to play an important role in rapid response to environmental stressors [27,29]. These results display the tight regulation of nitrogen assimilation in photosynthetic organisms and provide further evidence for the use of functional gene clusters in *P.* SENEW3 in rapid stress adaptation.

## Conclusion

Here, we examined the photophysiological and transcriptomic response of the picoeukaryote green alga *P*. SENEW3 to sublethal atrazine and glyphosate exposure. Atrazine exposure resulted in a significant reduction in maximum photosynthetic quantum yield; increased relative abundance of protein synthesis, carotenoid biosynthesis and carbon fixation gene transcripts; and decreased relative abundance of cell cycle and oxidase gene transcripts. This likely indicates the disruption of *P.* SENEW3’s cell cycle and a strong response to atrazine-induced oxidative stress, by increasing photoprotective compounds and modulating carbon fixation. Conversely, sublethal glyphosate exposure resulted in a decrease in *F_m_* and an increase in transcripts involved with the photoprotection of PSII antenna complex, indicating a likely response to potential photodamage by increasing NPQ. The glyphosate response also involved the enhanced transcription of glycolytic enzymes and altered expression of fatty acid biosynthesis and degradation genes. This could indicate a metabolic shift to utilize carbon reserves and fatty acid catabolism for energy generation; however, this may also just reflect a modification of the cellular redox state. Furthermore, glyphosate treatment resulted in lowered expression of protein synthesis, aa synthesis and shikimate pathway enzyme genes, which likely is a direct response to the compound’s mode of action. Finally, both treatments resulted in the decreased transcription of a nitrate assimilation cluster, which highlights the role of functional gene clusters in rapid environmental stress response in *P.* SENEW3. The results of this study demonstrate the utility of this picoeukaryote as a simple model for exploring the impacts of agricultural practices on photosynthetic marine organisms, allowing rapid assessment of the biological impact of anthropogenic pollutants, such as herbicides, on this important group of primary producers.

## Supplementary material

10.1099/mgen.0.001402 Uncited Supplementary Material 2.

## Abbreviations

EPSP: 5-enolpyruvylshikimate-3-phosphate synthase
FCM: flow cytometry
FSC-A: forward scatter area
log2 FCR: log2 fold change ratio
NPQ: non-photochemical quenching
PAM: pulsed amplitude modulation
*P*. SENEW3: *Picochlorum* sp. SENEW3
PSII: photosystem II
ROS: reactive oxygen species
TCA: tricarboxylic acid
